# William Conrad Wile

**Published:** 1913-04

**Authors:** 


### OBITUARIES.

---

William Conrad Wile, N. Y. University, 1870, died in Danbury, Conn., February 21, aged 66. He was a Civil War veteran and was the editor of the New England Medical Monthly for many years.

